# Dilated cardiomyopathy caused by mutation of the *PNPLA2* gene: a case report and literature review

**DOI:** 10.3389/fgene.2024.1415156

**Published:** 2024-07-25

**Authors:** Shuai Wang, Sha Wu, Daoquan Peng

**Affiliations:** Department of Cardiovascular Medicine, Second Xiangya Hospital of Central South University, Changsha, Hunan, China

**Keywords:** neutral lipid storage disease, cardiomyopathy, *patatin-like phospholipase domain-containing protein 2*, adipose triglyceride lipase, autosomal recessive disease, case report

## Abstract

Deficiency of adipose triglyceride lipase (ATGL) due to mutation in *PNPLA2* causes neutral lipid storage disease with myopathy (NLSDM), an autosomal recessive disorder (MIM: #610717). NLSDM patients are mainly affected by progressive myopathy, cardiomyopathy, and hepatomegaly. Cardiac involvement was reported in 40%–50% of NLSDM patients. Patients with cardiac involvement have adult-onset progressive heart failure, mimicking dilated or hypertrophic cardiomyopathy. The clinical characteristics, genotype–phenotype correlation, and prognosis of cardiomyopathy secondary to *PNPLA2* mutation are not understood. We reported two male patients carrying a homozygous splicing mutation NM_020376.4 (c.757 + 1G>T) in *PNPLA2,* presenting with severe dilated cardiomyopathy and mild skeletal muscle involvement. Through the literature review, the ECG and imaging features and the prognosis of 49 previously reported cases of cardiomyopathy caused by the *PNPLA2* mutation were summarized. This study suggests that NLSDM should be considered a cause of cardiomyopathy, especially in those with elevated creatine kinase (CK) levels, regardless of whether symptoms such as muscle weakness or atrophy are present.

## 1 Introduction

Fatty acids (FAs) provide > 70% of “fuel” for the heart (Lopaschuk et al., 2021). Exogenously imported FAs can be either oxidized or stored in cardiomyocytes as triglycerides (TAGs). A precisely regulated balance between FA uptake, TAG synthesis, TAG hydrolysis, and FA oxidation is the prerequisite for effective cardiac FA metabolism and proper heart functioning (Goldberg et al., 2012). Increasing evidence suggests that myocardial cytosolic lipolysis, in particular, adipose triglyceride lipase (ATGL) activity, plays an important role in the development of heart failure (HF) (Kintscher et al., 2020).

ATGL, encoded by gene *patatin-like phospholipase domain-containing protein 2 (PNPLA2)* in humans, catalyzes the first step of the hydrolysis of cytoplasmic TAG that is stored in lipid droplets (LDs). Patients carrying *PNPLA2* mutations that resulted in the loss or decreased function of ATGL develop neutral lipid storage disease with myopathy (NLSDM), an autosomal recessive disorder. People with NLSDM have abnormal TG storage in many tissues, particularly in the skeletal muscle and myocardium (Missaglia et al., 2019). The clinical phenotypes of NLSDM vary from elevated creatine kinase (CK) levels and progressive skeletal myopathy with or without cardiomyopathy to the rarely reported isolated cardiomyopathy without skeletal myopathy (Pennisi et al., 2017; Rao et al., 2019; Zhang et al., 2019; Fu et al., 2023). Currently, almost 130 NLSDM patients with more than 60 different *PNPLA2* mutations have been described (Missaglia et al., 2022). Although cardiac steatosis and cardiomyopathy in NLSDM patients have been described in the case report, the number is limited, and large cohort studies are lacking. The clinical characteristics, genotype–phenotype correlation, and prognosis of cardiomyopathy secondary to *PNPLA2* mutation are not understood.

In this study, we reported two male patients with NLSDM presenting with severe dilated cardiomyopathy and mild skeletal myopathy and carrying a homozygous splicing mutation in *PNPLA2*. We also reviewed the clinical and genetic characteristics of cases with cardiomyopathy due to *PNPLA2* mutation in the literature.

## 2 Case presentation

### 2.1 Case 1

#### 2.1.1 Clinical characteristics

The 19-year-old male was born from to healthy non-consanguineous parents. Since childhood, he had reduced exertional capacity compared with his peers but had not been medically evaluated. At the age of 18 years, the patient had exertional dyspnea when walking quickly and had to stop to rest. It was not until a month ago that these symptoms markedly worsened, and he began to suffer from paroxysmal nocturnal dyspnea, which led him to seek medical attention at the hospital.

His general physical examination was unremarkable. The heart was enlarged with no signs of fluid overload. Muscle wasting in upper and lower limbs was noted. The strength examination was normal. Reflex, cognition, coordination, sensation, and ambulation were normal. The patient was able to stand from a squatting position.

#### 2.1.2 Diagnostic assessment

**FIGURE 1:**
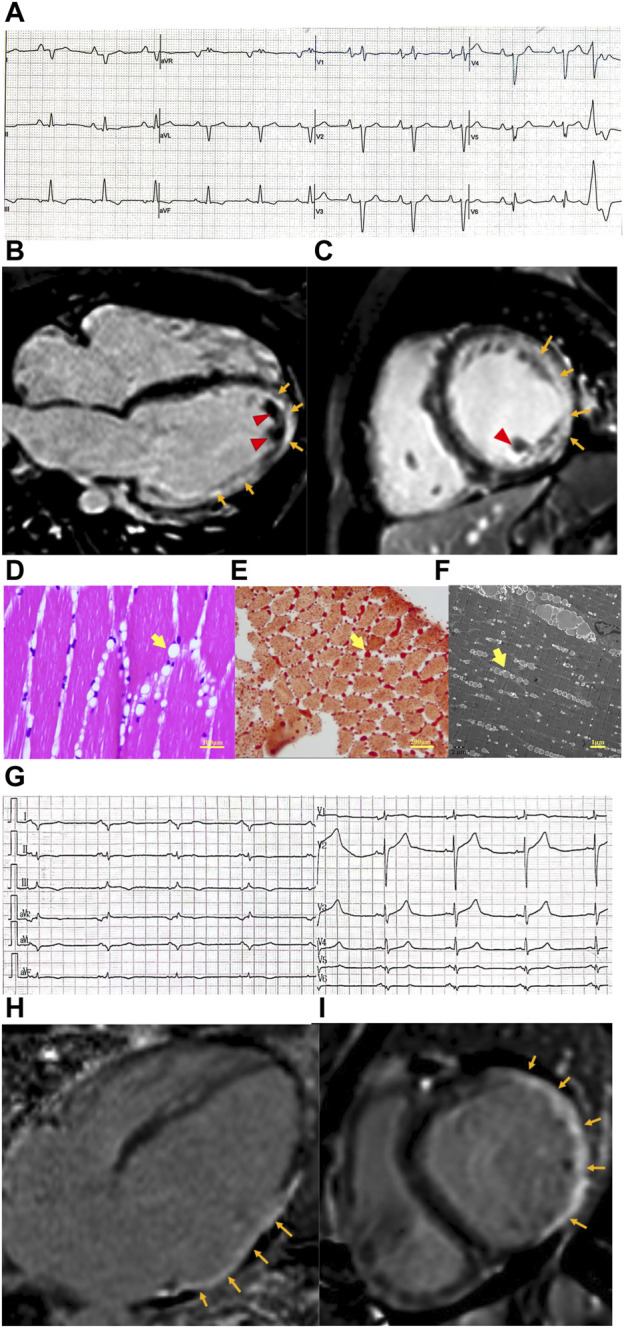
Clinical features of patients with cardiomyopathy and *PNPLA2* mutation. **(A)** Electrocardiogram of case 1 revealed poor R wave progression in precordial leads and Q wave progression in I, aVL lead, and PVC. **(B,C)** Cardiac magnetic resonance of case 1. Four-chamber view **(B)** and short axis view **(C)** reveal extensive subepicardial late gadolinium enhancement in the lateral wall, which extends to the epical segment (orange arrow), and mural thrombosis was observed (red arrow head). **(D–F)** Skeletal muscle biopsy obtained from case 1. **(D)** Hematoxylin–eosin **(E–H)** staining of skeletal muscle shows variation in myofiber size and cytoplasmic lipid vacuoles (yellow arrow head). **(E)** Lipid vacuoles in myocytes are stained by Oil Red O (yellow arrow head). **(F)** Transmission electron microscopy shows lipid droplets (yellow arrow head) located between the sarcomeric filaments and under the plasma membranes. **(G)** Electrocardiogram of case 1 revealed poor R wave progression in precordial leads, Q wave progression in I, aVL, and V_5,6_ leads, and low voltage in the limb lead. **(H,I)** Cardiac magnetic resonance of case 1. Four-chamber view **(H)** and short axis view **(I)** revealed transmural delayed gadolinium enhancement in the lateral wall (orange arrow).

Laboratory studies showed elevated troponin T (90.1 pg/mL ↑, normal value <14 pg/mL) and NT-proBNP (3,147 pg/mL ↑, normal value <125 pg/mL). The electrocardiogram showed poor R wave progression in V_1–5_ and Q wave progression in leads V_6_, I, and aVL (Figure 1A). Echocardiogram and cardiac magnetic resonance (CMR) imaging revealed a severely dilated left ventricle and impaired left ventricular function with akinesis of the inferolateral wall. An apical mural thrombus was noted. The left ventricular ejection fraction (LVEF) was 22%. Late gadolinium enhancement (LGE) imaging showed transmural enhancement in the inferolateral and apical segments. Coronary angiography was normal (Figures 1B, C).

The CK level was 757 U/L↑ (normal range 50–310 U/L). Electromyography (EMG) showed prominent myopathy affecting both distal and proximal muscle fibers. Nerve conduction studies were normal. A muscle biopsy of the gastrocnemius muscle was performed, and histologically, the muscle had diffused vacuolar change. There were no necrotic, regenerating, ragged red, or split fibers. No significant inflammatory infiltrates were noted. Oil red O showed a marked increase in sarcoplasmic lipid content. Micrographs from ultrastructural studies showed an extensive accumulation of variably sized sarcoplasmic and subsarcolemmal lipid droplets (Figures 1D–F).

#### 2.1.3 Genetic diagnosis

**FIGURE 2:**
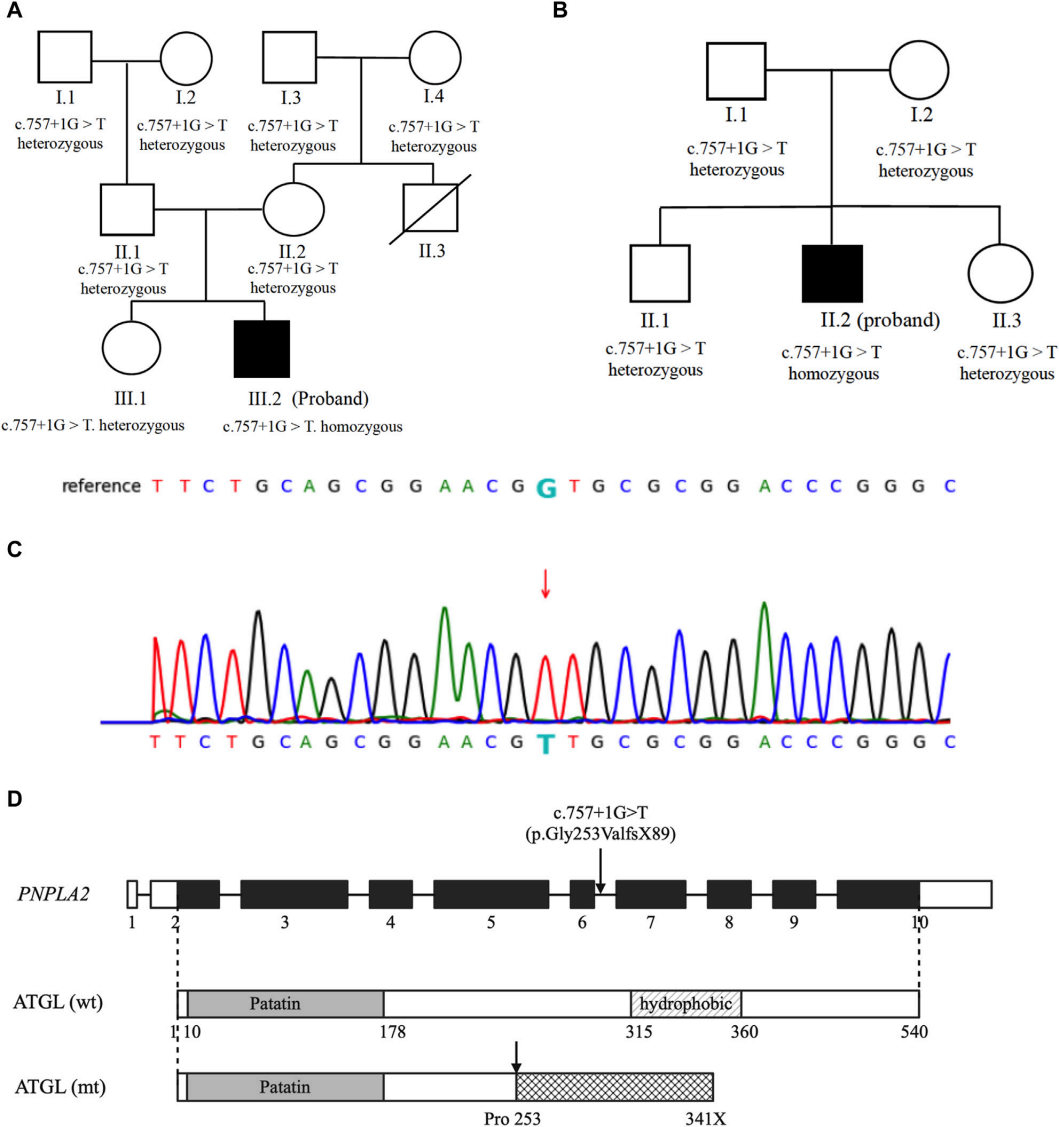
Genetic analysis: **(A,B)** genetic analysis of the probands and their family members. Probands are represented by black symbols. **(C)** Partial sequence of the *PNPLA2* gene in our patients. The arrow marks the mutation site. **(D)** Schematic representation of the native and mutated ATGL protein.

Whole-genome sequencing of the patient revealed homozygosity for the c.757 + 1G>T mutation in the *PNPLA2* gene. Screening for the disease phenotype and verification of the mutation were conducted among the first-degree relatives of the patient, revealing that his parents and sister also carry this variant, all being heterozygous without heart or muscle involvement, supporting the notion that the gene mutation leads to an autosomal recessive inherited disease (Figure 2A).

The patient received optimal, guideline-directed medical therapy. In the following 12 months, he was hospitalized three times for decompensated heart failure. He was referred for an assessment to receive a heart transplant and was placed on the heart transplant list.

### 2.2 Case 2

#### 2.2.1 Clinical characteristics

A 31-year-old male recently sought medical attention for breathlessness following mild physical activity, a condition accompanied by notable swelling in both of his legs and abdominal distension. Six years ago, he began to experience an unusual sense of fatigue and a decline in his overall physical strength. As a computer programmer, he initially dismissed these symptoms as the consequence of a demanding work schedule and insufficient physical activity. The patient was born to two healthy consanguineous parents, who were of the Hmong ethnicity.

The physical examination showed that the cardiac silhouette was enlarged. Diminished S1 and a 2/6-grade systolic blowing murmur could be heard at the apex. The neuro–muscular system examination showed normal limb muscle strength and tendon reflexes without obvious muscle atrophy.

#### 2.2.2 Diagnostic assessment

The laboratory test showed elevated NT-proBNP (8,240 pg/mL, normal value <125 pg/mL) and troponin T levels (58.4 pg/mL, normal value <14 pg/mL). The electrocardiogram was notable for the Q wave in V_5,6_, I, and aVL leads. Low voltage in the limb lead was also observed (Figure 1G). Echocardiography and cardiac MRI revealed significant dilatation of the left heart (left ventricular end-diastolic diameter [LVEDd], 72 mm; left atrial end-systolic diameter [LAS], 51 mm), diffusely reduced wall motion, and markedly decreased left ventricular systolic function with an LVEF of 39%. CMR-LGE showed transmural enhancement of the lateral wall (Figures 1H, I). Coronary computed tomography angiography (CTA) did not reveal any abnormalities.

The laboratory test showed that the patient had elevated CK levels (1,516 U/L, normal range 50–310 U/L). Further EMG results were compatible with the myopathy of the upper extremities. A peripheral blood smear showed the presence of vacuoles in the cytoplasm of peripheral blood leukocytes.

#### 2.2.3 Genetic diagnosis

Whole-genome sequencing of the patient revealed homozygosity for the c.757 + 1G>T mutation in the *PNPLA2* gene. Family analysis among the first-degree relatives revealed that the patient’s parents, sister, and brother are all heterozygous carriers of this variant, and none of them exhibit a similar disease phenotype (Figure 2B).

The patient was treated with optimal, guideline-directed medical therapy and remained stable for the following 6 months, according to the New York Heart Association (NYHA) functional class III.

## 3 Discussion

We presented two adult male cases of NLSDM with dilated cardiomyopathy and mild skeletal muscle symptoms or asymptomatic CK elevation. NLSDM is caused by mutation in *PNPLA2*, which encodes major enzymes catalyzing cytosolic lipolysis ATGL. ATGL deficiency causes lipid accumulation in multiple organs, primarily skeletal muscle and myocardium; the liver and pancreas can also be affected (Missaglia et al., 2019). Although NLSDM typically manifests as muscle weakness in young adults, 40%–50% of NLSDM patients exhibit cardiac dysfunction later in the course of the disease (Pennisi et al., 2017; Zhang et al., 2019; Fu et al., 2023).

ATGL deficiency in the heart leads not only to impaired intracellular TG hydrolysis and lipotoxicity due to TG accumulation in cardiomyocyte but also to a striking defect in the activation of the transcription factor peroxisome proliferator-activated receptor-α (PPARα) and reduced mitochondrial biogenesis and function (Haemmerle et al., 2011). In previous reports, the majority presented with dilated cardiomyopathy (DCM), although hypertrophic cardiomyopathy (HCM) and arrhythmogenic cardiomyopathy (ACM) had also been reported (Akiyama et al., 2007; Hirano et al., 2008; Kobayashi et al., 2008; Ohkuma et al., 2008; Hirano et al., 2014; Kaneko et al., 2014; Higashi et al., 2015; Muggenthaler et al., 2016; Pasanisi et al., 2016; Rao et al., 2019; Rajani et al., 2020). For the majority of patients, muscle weakness is the initial clinical manifestation, and cardiac dysfunction appears later in the course of the disease. However, there are cases where heart failure and arrhythmias are the main clinical manifestations, with mild skeletal muscle involvement or asymptomatic elevated CK levels (Kobayashi et al., 2008; Kaneko et al., 2014; Higashi et al., 2015; Rao et al., 2019; Rajani et al., 2020). Cardiomyopathy related to *PNPLA2* loss-of-function variants could result in a poor prognosis. In 51 NLSDM patients with cardiac involvement, 5 (9.8%) died of heart failure and 5 (9.8%) received a heart transplant (Higashi et al., 2015; Rajani et al., 2020) (Supplementary Table S1).

Previous case reports on NLSDM have focused more on the distribution and imaging characteristics of myopathy, with less description of the electrocardiographic and cardiac imaging features of myocardial involvement. We summarized the clinical characteristics of previously reported NLSDM patients with cardiac dysfunction, as well as the two cases we presented (Supplementary Table S1, S2). Cases with detailed descriptions of electrocardiogram, echocardiogram, or cardiac magnetic resonance features were analyzed. ECG findings include poor R wave progression in the precordial leads (3/51, 5.9%), low voltage in the limb leads (2/51, 3.9%), and Q wave in the lateral leads (3/51, 5.9%). Frequent ventricular premature beats and ventricular tachycardia were found in 5/51 (9.8%) of the patients. Echocardiography and cardiac magnetic resonance imaging (MRI) revealed diffuse hypokinesis of the ventricular walls with a reduced ejection fraction, which was reported in 12/51 (23.5%) patients. Left ventricular dilation and left ventricular hypertrophy were found in 7/51 (13.7%) patients, while biventricular dilation and isolated right ventricular dilation were found in 1/51 (2.0%) patients. Hypokinesis of the inferolateral wall with the corresponding transmural delayed enhancement of this segment on the gadolinium late enhancement is a common feature, which was observed in 5/51(9.8%) patients. Cardiac death or heart transplantation was recorded in 11/51 (21.6%) of patients with cardiomyopathy and the *PNPLA2* mutation (Supplementary Table S2). Although not routinely used, the ECG-gated 64-slice CT scan and washout rate of iodine-123-β-methyl iodophenyl-pentadecanoic (BMIPP) acid in the myocardial scintigram were reported to specifically identify myocardial TG deposition and impaired long-chain fatty acid (LCFA) metabolism and assist in the diagnosis of cardiomyopathy due to the *PNPLA2* mutation (Higashi et al., 2015; Hirano et al., 2015; Chen et al., 2022; Miyauchi et al., 2023).

**FIGURE 3:**
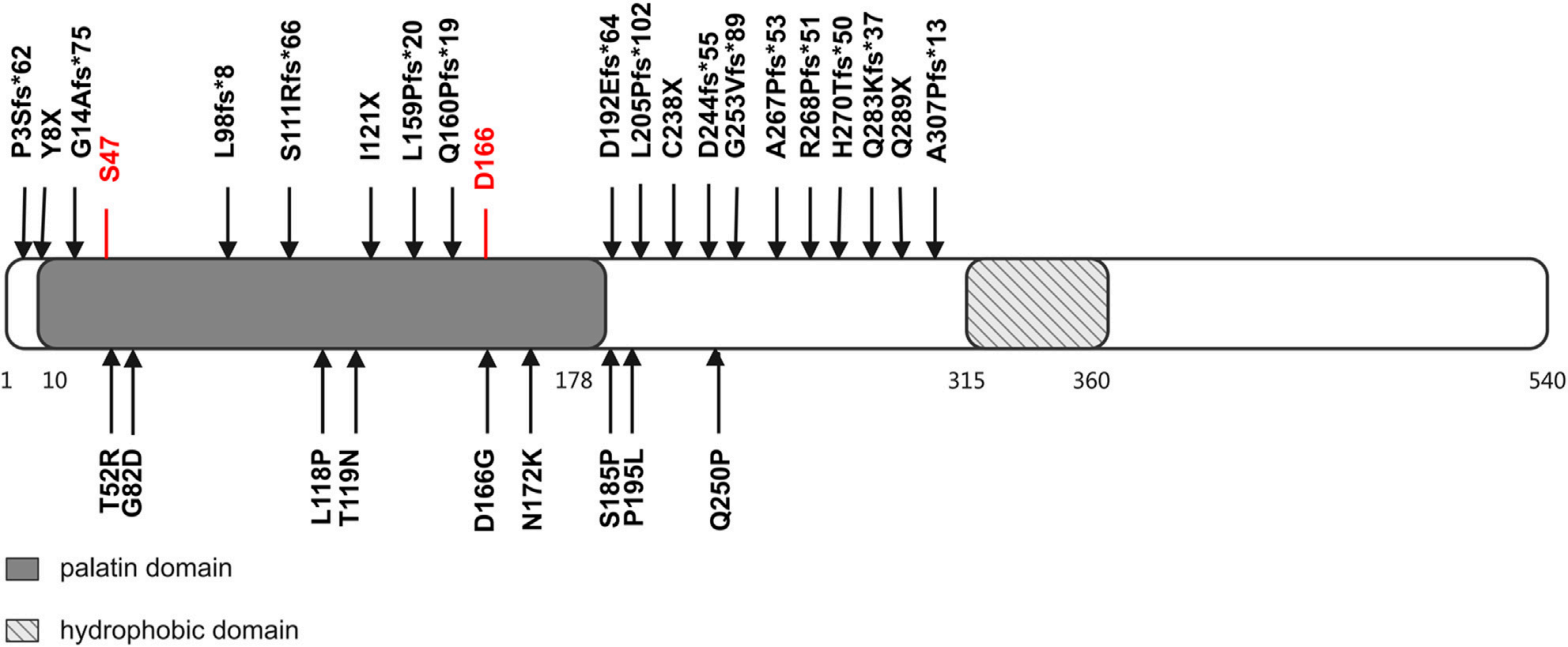
Structural domains of ATGL protein and mutations identified in patients with cardiomyopathy and *PNPLA2* mutation. The ATGL protein consisted of 504 amino acids. There are two functional domains/regions: (1) the patatin domain, which contains a catalytic site (S47 and D166), and (2) a hydrophobic region in the C-terminal, which takes part in binding with lipid droplets. Predicted ATGL protein changes of reported *PNPLA2* mutations are listed.

In NLSDM patients, a spectrum of phenotypes has been observed, ranging from relatively asymptomatic CK elevation to the full expression of severe myopathy and cardiomyopathy. The evaluation of NLSDM symptoms suggests that many factors, including genotype, epigenetic factors, gender, and ethnicity, may modulate the clinical phenotype of this disorder. First, it has been proposed that whether the myocardium is involved, as well as the severity of cardiac involvement, may be related to the impact of the mutation sites on ATGL lipase activity (Fischer et al., 2007; Campagna et al., 2008; Kobayashi et al., 2008; Missaglia et al., 2019). Human ATGL is a 504-amino acid protein, which harbors a patatin domain and contains a catalytic dyad consisting of Ser47 and Asp166 (Grabner et al., 2021). The carboxy-terminal half of the enzyme contains a hydrophobic stretch (325–360 residues), which is required for LD binding and TG hydrolysis (Schweiger et al., 2008). Previously reported findings provide evidence that NLSDM patients who carry missense mutations manifest a mild disease phenotype, especially cardiac symptoms, with the exception of patients who carry a missense mutation that disrupts the ATGL catalytic site (Coassin et al., 2010). However, in 51 patients with NLSDM-associated cardiomyopathy, both frameshift and missense mutations were identified (Figure 3). It is difficult to summarize the relationship between genotype and phenotype based on this limited number of patients and the lack of ATGL enzymatic activity analysis. Future molecular and functional analyses of *PNPLA2* mutations are needed to clarify the variation in the clinical expression of the syndrome. Second, a wide range of phenotypic variability has been found in patients carrying the same mutation, both in terms of target organs and severity of the disease, suggesting that epigenetic factors may affect the disease phenotype (Missaglia et al., 2015; Pennisi et al., 2017). Furthermore, estrogen appears to have a protective effect in patients carrying the *PNPLA2* mutation since heart failure has been reported in almost 20% of NLSDM female patients and 55% of male patients, although the frequency of *PNPLA2* mutations that cause a lack of ATGL protein production or expression of truncated proteins is similar in men and women (Higashi et al., 2015; Missaglia et al., 2017). Lastly, ethnic disparities in cardiac involvement were reported. In a cohort of Italian patients with NLSDM, mild cardiac involvement was observed in several patients, which did not, however, require therapies other than antihypertensive treatment. This is different from that observed in the subjects from Japan, in whom cardiac involvement seems to be the main clinical feature and often leads to heart transplantation or cardiac death (Hirano et al., 2008; Ohkuma et al., 2008; Kaneko et al., 2014; Higashi et al., 2015).

c.757 + 1G>T was previously reported in a small case series of Hmong patients of Southeastern Asian origin and in a large cohort of NLSDM patients from China as the hotspot mutation (Lin et al., 2012; Laforet et al., 2013; Latimer et al., 2018; Tan et al., 2018; Zhang et al., 2019). The mutation disrupts the splicing donor site of introns 5–6 and is predicted to result in the complete inclusion of introns 5–6 (106 bp) into the mRNA (p.Gly253ValfsX89) (Figures 2C, D). To date, there have been 24 cases of homozygous patients carrying c.757 + 1G>T, including 15 females and 9 males, with an average onset age of 29.7 ± 9.4 years. A trend of a later onset of disease in females than males was observed, although the difference is not significant (females vs. male: 31 ± 10.0 vs. 28 ± 8.5 years, *p* > 0.05). In terms of the presence of myocardial involvement, there is heterogeneity among patients carrying the mutation, with 45.8% (11/24) of cases accompanied by cardiomyopathy. In addition, one case was reported to have isolated cardiac involvement without skeletal muscle affection (Supplementary Table S3). The impact of this splice mutation of *PNPLA2* on the enzymatic activity of the ATGL and LCFA metabolism in myocardial cells is not known.

## 4 Conclusion

In conclusion, we describe NLSDM as a rare cause of DCM. These two patients have cardiac involvement as the main clinical manifestation, while the symptoms of skeletal muscle involvement are mild or only accompanied by elevated CK levels without muscle weakness. Our report suggests that for patients with cardiomyopathy accompanied by elevated CK levels, the possibility of neutral lipid storage disease should be considered, and the *PNPLA2* gene should be considered for inclusion in cardiomyopathy genetic panels.

## Data Availability

The original contributions presented in the study are included in the article/Supplementary Material; further inquiries can be directed to the corresponding author.
