# Supplementary figures and images for: Dilated cardiomyopathy caused by mutation of the PNPLA2 gene: a case report and literature review
Source: Front Genet. 2024 Jul 25;15:1415156. doi: 10.3389/fgene.2024.1415156 (PMC11306180; doi:10.3389/fgene.2024.1415156)

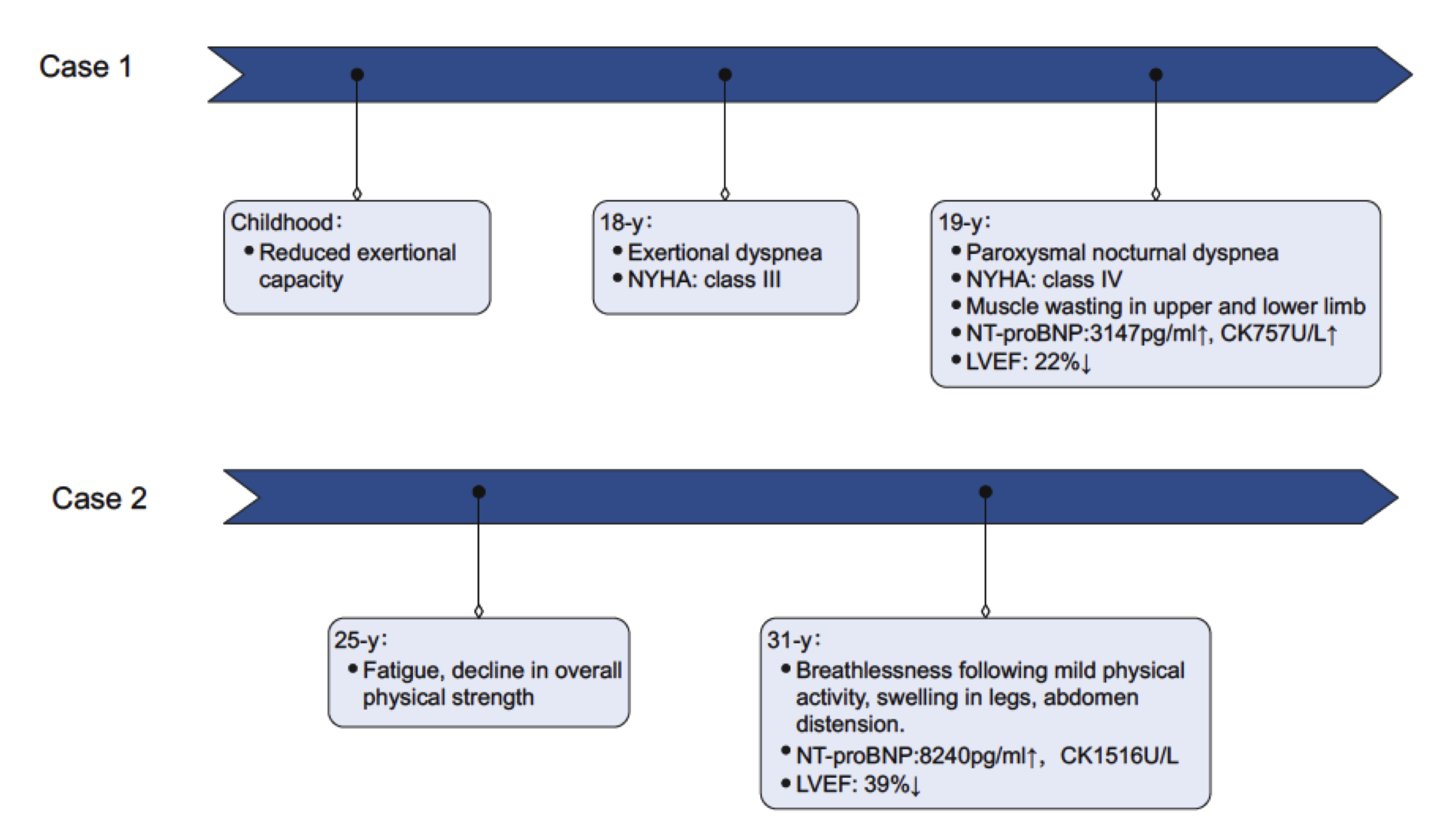

Supplement: Supplementary file 2 [file Image1.TIFF]
