# Supplementary material for: Dilated cardiomyopathy caused by mutation of the PNPLA2 gene: a case report and literature review
Source: Front Genet. 2024 Jul 25;15:1415156. doi: 10.3389/fgene.2024.1415156 (PMC11306180; doi:10.3389/fgene.2024.1415156)
Supplement: Supplementary file 5 [file Table4.DOCX]

**The CARE Checklist**

**1. Title – The diagnosis or intervention of primary focus followed by the words “case report”.**

Dilated Cardiomyopathy Caused by Mutation of the PNPLA2 Gene: A Case Report and Literature Review

**2. Key Words – 2 to 5 key words that identify diagnoses or interventions in this case report (including "case report").**

Neutral lipid storage disease, Cardiomyopathy, PNPLA2, ATGL, autosomal recessive disease, case report.

**3. Abstract – (structured or unstructured)**

**Introduction – What is unique about this case and what does it add to the scientific literature? √**

**The patient’s main concerns and important clinical findings.** √

**The primary diagnoses, interventions, and outcomes.** √

**Conclusion – What are one or more “take-away” lessons from this case report?** √

Deficiency of adipose triglyceride lipase (ATGL) due to mutation in PNPLA2 cause autosomal recessive disease neutral lipid storage disease with myopathy (NLSDM) (MIM: #610717). NLSDM patients with cardiac steatosis and cardiomyopathy are rarely reported, the clinical characteristics, genotype-phenotype correlation, and prognosis of cardiomyopathy secondary to PNPLA2 mutation is not understood. We reported two male patients carrying homozygous splicing mutation NM_020376.4 (c.757+1G>T) in PNPLA2, presenting with severe dilated cardiomyopathy and mild skeletal muscle involvement. Through literature review, the ECG and imaging feature as well as prognosis of 37 cases of cardiomyopathy caused by PNPLA2 mutation were summarized. This study suggest NLSDM should be considered as cause of cardiomyopathy, especially those with elevated CK levels regardless of whether symptoms such as muscle weakness or atrophy are present.

**4. Introduction**

**– Briefly summarizes why this case is unique and may include medical literature references.** Clinical phenotypes of patients carrying loss-of-function mutations in the *PNPLA2* vary from elevated CK levels, progressive skeletal myopathy with or without dilated cardiomyopathy to the rarely reported isolated cardiomyopathy without skeletal myopathy. Currently, approximately 100 people with more than 30 different mutations of this gene have been identified.(Grabner et al., 2021) Although some case reports and case series reported affected individual with cardiac steatosis and cardiomyopathy, these is lack of large cohort studies. The clinical characteristics, genotype-phenotype correlation, and prognosis of cardiomyopathy secondary to *PNPLA2* mutation is not understood. (See main manuscript “Introduction” section)

**5. Patient Information**

**De-identified patient specific information.** (See main manuscript “2 Case presentation”-“clinical characteristics” )

**Primary concerns and symptoms of the patient.** (See main manuscript “2 Case presentation”-“clinical characteristics” )

**Medical, family, and psychosocial history including relevant genetic information.** (See main manuscript “Case presentation”——“clinical characteristics” )

**Relevant past interventions and their outcomes.** (See main manuscript “Case presentation”——“clinical characteristics” )

**6.Clinical Findings**

**– Describe significant physical examination (PE) and important clinical findings.** (See main manuscript “Case presentation”——“clinical characteristics” )

**7. Timeline –** **Historical and current information from this episode of care organized as a timeline (figure or table).** The timeline figure has been uploaded as supplement figure.

**
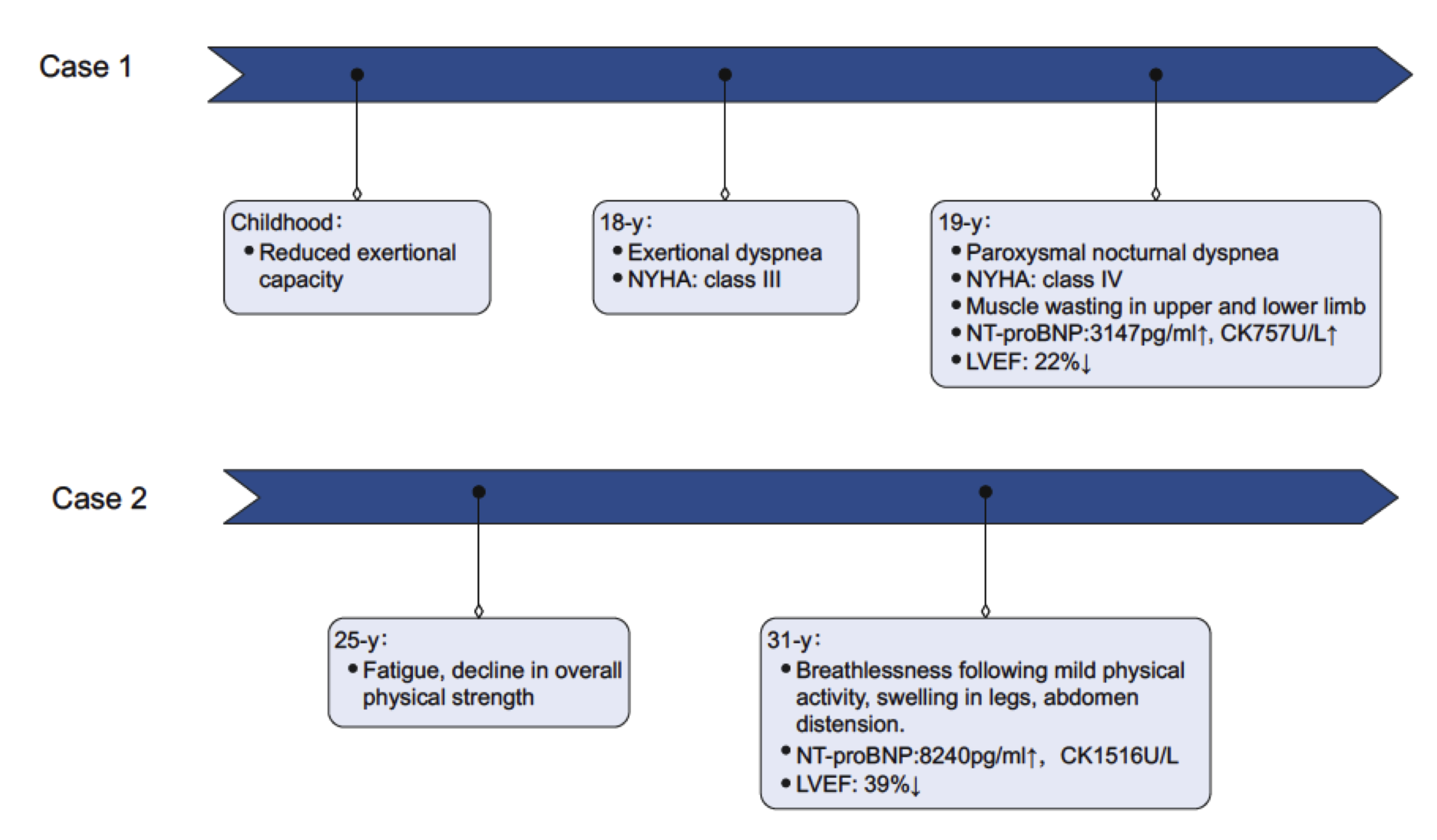
**

**8. Diagnostic Assessment**

**Diagnostic methods (PE, laboratory testing, imaging, surveys).**  See main manuscript “Case presentation”——“Diagnostic assessement”

**Diagnostic challenges.** NA

**Diagnosis (including other diagnoses considered).** See main manuscript “Case presentation”——“Genetic diagnosis”

**Prognostic characteristics when applicable.** NA

**9. Therapeutic Intervention**

**Types of therapeutic intervention (pharmacologic, surgical, preventive).** Optimal pharmacotherapy for heart failure.

**Administration of therapeutic intervention (dosage, strength, duration).**

For Case 1: Sacubactril valsartan 50mg bid po. (1 month) – 100mg bid po. (1 month) – 200mg bid po. (1 week) — 150mg bid po. (10 months). Dapagliflozin 10mg qd po. (12 months). Bisoprolol 2.5mg qd po. (1 month) — 5mg qd po. (11 months). Spironolactone 20mg qd po. (12 months). Furosemide 20mg qd po. (12 moths). Warfarin 2.5mg qd po. (12 months). Digoxin 0.25mg qd po. (12 months).

Case 2: Sacubacrtil valsartan 100mg bid po. (1 month), 200mg bid po. (5 months). Dapagliflozin 10mg qd po. (12 months). Bisoprolol 5mg qd po. (6 months). Spironolactone 20mg qd po. (6 months).

**Changes in therapeutic interventions with explanations.** Despite receiving optimized treatment for heart failure with medications, the patient's (case 1) cardiac function continued to deteriorate, awaiting a heart transplant.

**10. Follow-up and Outcomes**

**Clinician- and patient-assessed outcomes if available.** Patient 1 were hospitalized 3 times for decompensated heart failure during the 1-year follow up and was placed on heart transplant list. Patient 2 remained stable in the 6-months follow up, with NYHA III.

**Important follow-up diagnostic and other test results.** NA

**Intervention adherence and tolerability. (How was this assessed?)** The 2 patients receive optimal pharmacotherapy for heart failure, takes medication regularly, and has routine follow-up visits at the outpatient clinic.

**Adverse and unanticipated events.** Patient 1 were hospitalized 3 times for decompensated heart failure during the 1-year follow up.

**11. Discussion**

**Strengths and limitations in your approach to this case.** We reported two male patients carrying homozygous splicing mutation NM_020376.4 (c.757+1G>T) in *PNPLA2,* presenting with severe dilated cardiomyopathy and mild skeletal muscle involvement. Both patients underwent detailed clinical evaluation and family screening, receiving optimal treatment for heart failure. Small number of cases and short follow-up period are limitations of the report.

**Discussion of the relevant medical literature.** See main manuscript “Discussion” section.

**The rationale for your conclusions.** √

**The primary “take-away” lessons from this case report (without references) in a one paragraph conclusion.**  In conclusion, we describe NLSDM as a rare cause of DCM. These two patients have cardiac involvement as the main clinical manifestation, while the symptoms of skeletal muscle involvement are mild or only accompanied by elevated CK levels without muscle weakness. Our report suggests that for patients with cardiomyopathy accompanied by elevated CK levels, the possibility of neutral lipid storage disease should be considered, and *PNPLA2* gene should be considered for inclusion in cardiomyopathy genetic panels.

**12. Patient Perspective – The patient should share their perspective on the treatment(s) they received.** Patient 1: “Current treatments for heart failure did not reverse the progression of the disease, I am waiting for heart transplants while facing the issues of insufficient donor supply and expensive treatment costs.” Patient 2: “The current treatment has alleviated the symptoms, although physical activity can lead to fatigue, basic daily activities and work can still be carried out.”

**13. Informed Consent – The patient should give informed consent. (If not, explain)** The patients have signed the informed consent.
